# Developing a High Velocity Dataset Quality Checking Pipeline

**DOI:** 10.23889/ijpds.v9i5.2741

**Published:** 2024-09-18

**Authors:** Hannah Davies, Daniel S Thayer, Muhammad A Elmessary, Alex-Ioan Coldea, Carys Jones, Lorna Evans, Alexander Makovics, Owen Howell-Wright, Lee Hughes

**Affiliations:** 1 Swansea University

## Objective

The volume and frequency of refreshed data within the [organisation] has increased significantly since the beginning of the COVID-19 pandemic. Therefore, a more efficient data quality (DQ) checking process was necessary.

## Approach

Having previously developed an automated DQ checking tool, the focus was on re-engineering the process of DQ task allocation and communication of results.

## Results

5 analysts were trained in DQ checking. A JIRA workflow tracks the management of data loading. When a dataset is ready for DQ, the Data Manager allocates a ticket to the DQ Lead who then allocates it onto one of the 5 analysts. Via a DQ Slack channel, the analyst is informed and acknowledges receipt of the task. On completion of DQ, the analyst updates the ticket and transfers it to the appropriate workflow stage. Passed DQ tickets are transferred to the Data Manager for data release, whereas failed ones are placed “On Hold”. The DQ Lead triages the issues and liaises with relevant parties for resolution, which may require data amendments. On receipt of amended data, the DQ ticket is transferred back to the queue and the analyst is notified to re-check the data.

## Conclusions

The team now complete a high volume of DQ checks efficiently. In 2023, 405 datasets, containing 1716 tables, were quality checked, with the initial DQ taking, on average, 2.6 days.

## Implications

The improved speed of DQ checking ensures projects access the latest available data whilst maintaining the expected DQ levels, integrity and reputation of the TRE.

